# Trainee perceptions of a group-based standardized patient training for challenging behavioral health scenarios in the United States

**DOI:** 10.3352/jeehp.2018.15.15

**Published:** 2018-06-11

**Authors:** Rachel A. Petts, Jeffrey D. Shahidullah, Paul W. Kettlewell, Kathryn Dehart

**Affiliations:** 1 Department of Psychiatry, Geisinger Health System, Danville, PA, USA; 2 Graduate School of Applied and Professional Psychology, Rutgers University, Piscataway, NJ, USA; 3 Department of Pediatrics, Geisinger Health System, Danville, PA, USA; Hallym University, Korea

Clinical teachers widely acknowledge the need to improve the instruction of medical trainees in behavioral health topic areas, as well as communication and relationship-building skills (hereafter referred to as communication skills) [1]. Despite this general consensus and appeals to promote changes in behavioral health curricula, programs continue to struggle to implement comprehensive and effective behavioral health educational experiences [2]. Currently, there is no agreement on ideal training methods in this content area that are practical to implement from a resource and time perspective and effective in preparing trainees to attain behavioral health communication competencies.

One promising option is the use of standardized patient (SP) encounters, a method commonly utilized in the training and evaluation of residents in traditional medical skills and general communication skills [3,4]. SPs are a realistic and active training experience in which a trainee engages in a one-on-one clinical encounter with a trained actor involving the diagnosis/management of a health condition. This real-time encounter allows for immediate performance feedback and, thus, has the potential to improve the retention and generalization of pertinent clinical skills [4]. However, SPs can also be time-/resource-intensive, particularly if intended for all trainees in several topic areas [4]. Not surprisingly, fewer than 8% of pediatric residency programs have reported using SPs for training purposes [5]. Thus, this project aimed to assess the acceptability and usefulness of a group-based SP training focused on challenging behavioral health scenarios that may prove more feasible to implement.

These group trainings with medical students and residents were considered a pilot quality improvement project to determine whether the approach would be useful or acceptable as a part of our pediatric residency behavioral health training curriculum. Trainees were invited to voluntarily attend 2 SP training sessions as a part of their typical educational curriculum. Paid actors (1 teenage female and 1 adult female) were recruited and trained by the hospital training site’s Standardized Patient Program. Two SP scenarios were developed by content experts (i.e., an attending physician, an attending clinical psychologist, and educational experts). A group format was used in both scenarios, similar to the “fishbowl method” of instruction [6], in which trainees observed and guided content experts during the encounter and contributed to the discussion.

The first SP scenario involved the delivery of unexpected bad news to a parent (i.e., a “Delivering Bad News” module, which involved a potential Down Syndrome diagnosis), in which an attending physician modeled communicating this information to a new parent with guidance from the group. “Time-outs” were taken as the scenario unfolded for the group to discuss the next appropriate communication choice. If the group decided that the delivery did not go as planned, a time-out was taken and another communication approach was utilized and evaluated.

The second scenario involved a postdoctoral psychology fellow and an attending clinical psychologist modeling ineffective and effective examples of utilizing motivational interviewing skills with a teenager who is using marijuana (i.e., a “Working with a Challenging Patient” module). After the ineffective role-play with the postdoctoral fellow, residents and medical students discussed what could have been improved based on their observations, and then a clinical psychologist modeled a more effective encounter. After both interviews, the trainees then practiced the strategies learned with a colleague using a “Working with a Challenging Patient” scenario or a personal behavioral goal of their own choosing.

A 6-item survey was developed by a psychology postdoctoral fellow, a psychologist, and a pediatrician to ensure the appropriateness of the content in terms of acceptability (Tables 1–3). Items 1–3 utilized a 6-point Likert-type scale to rate the level of agreement with each statement (1: strongly disagree to 6: strongly agree). Items 4–6 were open-ended. The raw data are available in Supplement 1.

The survey was administered after each training session. Informed consent to collect feedback regarding the training was obtained at this time. Prior to distributing the survey, trainees were verbally informed that completion of the survey was voluntary and their responses would be kept anonymous. Those who stayed to complete the survey provided implied consent to participate in data collection for the quality improvement project.

Descriptive statistics were utilized for the Likert-type items (1–3), while 2 of the open-ended items (4–5) were analyzed through qualitative classical content analysis. Two authors independently coded the open-ended responses for themes and then compared and discussed their respective themes and codes. Any discrepancies were reviewed and resolved to achieve concordance. Given the overlap in responses for item 6 (What other SP cases would you find helpful for your education?), frequency counts are provided for this item. The only demographic information collected was trainee status (i.e., medical student or residency year).

Twenty-seven trainees voluntarily completed the SP acceptability questionnaire across both training sessions. Forty-eight percent (n=13) were medical students. Nineteen percent were first-year residents (n= 5), 29% were second-year residents (n= 8), and a single fourthyear/chief resident replied (n= 1, 4%). No third-year residents were in attendance. Fifteen trainees attended the first SP training (“Delivering Bad News”), while 12 attended the second (“Working with a Challenging Patient”).

Tables 1 and 2 present descriptive data for items 1–3 for both trainings, indicating generally positive responses with regards to perceived benefit, interest in attending a future SP training, and relevance to their clinical work. Although no comparative analyses were performed, the “Working with a Challenging Patient” encounter exhibited slightly lower mean scores than the “Delivering Bad News” encounter (Table 1). Further, residents responded with slightly higher acceptability ratings than medical students on items 1 and 3 (Table 2).

Table 3 presents themes and relevant examples generated through classical content analysis for items 4–5. Themes relevant to both trainings included the non-judgmental aspect of the group experience and the opportunity to observe content experts modeling skills. Few suggestions were given to improve the training.

Proposed future SP training topics included: 6 suggestions for caregivers who are opposed to vaccinations, 6 related to death or terminal conditions, 5 for other risky behavior in adolescents (e.g., alcohol use), 3 for managing upset or aggressive patients, and 3 for health-related behavior change (e.g., weight).

The results from this project revealed that medical trainees found the group-based SP training experience beneficial to their training, relevant to their clinical work, and a program that they would attend again in the future. As a result, group-based SP trainings are being considered as a new training opportunity for all incoming pediatric residents in our program. This feedback may also inform other medical educators attempting to implement a behavioral health/communication curriculum. Training residents in this important area remains difficult due to numerous barriers, including the lack of time and attention devoted to this topic and the lack of opportunity for trainees to benefit from experienced practitioners modeling these skills [7]. The group-based approach to learning from SP encounters using an expert clinician modeling the skill, followed by trainees discussing or practicing the skills learned, allows for learning enhancement while avoiding the time and resource barriers that occur with traditional SP encounters.

This project has several limitations. Its findings are representative of a group of 27 trainees from Geisinger Medical Center in the northeastern United States; thus, caution is warranted when generalizing these results to other training sites or residency specialties. The acceptability survey was developed by the authors and has not been psychometrically validated. Relatedly, kappa coefficients were not computed for the qualitative data. There could also have been order effects for trainees who attended both sessions. Finally, this project was not designed to evaluate the relative effectiveness of this training approach for learning enhancement.

In sum, the high acceptability ratings from medical trainees for this relatively feasible training experience suggests that future research and implementation may be warranted. Future research should address the degree to which the training experience improves other key outcomes, including patient ratings of clinical improvement or satisfaction with the healthcare encounter.

## Figures and Tables

**Table 1.** Descriptive statistics for the acceptability questionnaire in the combined sample and divided by training content

Item	Combined (n=27)		“Delivering Bad News” (n=15)		“Working with a Challenging Patient” (n=12)	
	Mean±SD	Range	Mean±SD	Range	Mean±SD	Range
1. My experience with the standardized patient was beneficial to my training in delivering behavioral health care.	5.56 ± 0.51	5–6	5.67 ± 0.49	5–6	5.42 ± 0.51	5–6
2. I would attend another standardized patient training in the future.	5.48 ± 0.51	5–6	5.6 ± 0.51	5–6	5.33 ± 0.49	5–6
3. The content of the standardized patient training was relevant to my work as a clinician.	5.70 ± 0.47	5–6	5.8 ± 0.41	5–6	5.58 ± 0.51	5–6

SD, standard deviation.

**Table 2.** Descriptive statistics for the acceptability questionnaire divided by training level

Item	Medical students (n=13)		Residents (n=14)	
	Mean±SD	Range	Mean±SD	Range
1. My experience with the standardized patient was beneficial to my training in delivering behavioral health care.	5.46 ± 0.52	5–6	5.64 ± 0.50	5–6
2. I would attend another standardized patient training in the future.	5.62 ± 0.51	5–6	5.36 ± 0.50	5–6
3. The content of the standardized patient training was relevant to my work as a clinician.	5.62 ± 0.51	5–6	5.79 ± 0.43	5–6

SD, standard deviation.

**Table 3.** Themes and examples from open-ended items

Item	Themes		Examples
4. What did you like most about the standardized patient training?	“Delivering Bad News”		
		Modeling from expert	“Interaction with attendings & their experience and expertise”
		Non-judgmental	“Non-threatening – I didn’t have to be in the hot seat”
		Collective/team-based participation	“Liked that it was a group sessions – helped to hear how different people would respond to the situation”
		Incremental steps	“It was useful to pause and discuss ideas of how to proceed”
	“Working with a Challenging Patient”		
		Contrasting bad vs. good encounter	“I liked seeing a ‘bad’ encounter which is similar to what we all do and then an effective one”
		Modeling from expert	“Example of how to connect with a patient during motivational interviewing”
		Interactive/realistic	“Educational and realistic”
		Discussion questions	“The case scenarios and the discussion afterwards”
		Role play practice	“Being able to practice talking and asking the right questions”
		Non-judgmental	“It’s not threatening. Not intimidating”
5. What aspects of this training could be improved?	“Delivering Bad News”		
		More time/repetition	“It would be beneficial to have more time as it felt rushed as it got to the end”
		More initial guidance at the beginning	“Allowing a pre-run to more effectively model communication”
		Nothing	“None, it was great”
	“Working with a Challenging Patient”		
		Nothing	“Good presentation and testing our skills with practice cases”

## Supplementary material

Supplement 1. Data files are available from https://doi.org/10.7910/DVN/DQQ39A.
